# SOUP-GAN: Super-Resolution MRI Using Generative Adversarial Networks

**DOI:** 10.3390/tomography8020073

**Published:** 2022-03-24

**Authors:** Kuan Zhang, Haoji Hu, Kenneth Philbrick, Gian Marco Conte, Joseph D. Sobek, Pouria Rouzrokh, Bradley J. Erickson

**Affiliations:** 1 Department of Radiology, Mayo Clinic, Rochester, MN 55905, USA; zhang.kuan@mayo.edu (K.Z.); philbrick.kenneth@mayo.edu (K.P.); conte.gianmarco@mayo.edu (G.M.C.); sobek.joseph@mayo.edu (J.D.S.); rouzrokh.pouria@mayo.edu (P.R.); 2 Department of Computer Science & Engineering, The University of Minnesota, Minneapolis, MN 55455, USA; huxxx899@umn.edu

**Keywords:** super-resolution, magnetic resonance imaging (MRI), generative adversarial networks (GAN), 3D perceptual loss, medical imaging interpolation, deep learning

## Abstract

There is a growing demand for high-resolution (HR) medical images for both clinical and research applications. Image quality is inevitably traded off with acquisition time, which in turn impacts patient comfort, examination costs, dose, and motion-induced artifacts. For many image-based tasks, increasing the apparent spatial resolution in the perpendicular plane to produce multi-planar reformats or 3D images is commonly used. Single-image super-resolution (SR) is a promising technique to provide HR images based on deep learning to increase the resolution of a 2D image, but there are few reports on 3D SR. Further, perceptual loss is proposed in the literature to better capture the textural details and edges versus pixel-wise loss functions, by comparing the semantic distances in the high-dimensional feature space of a pre-trained 2D network (e.g., VGG). However, it is not clear how one should generalize it to 3D medical images, and the attendant implications are unclear. In this paper, we propose a framework called SOUP-GAN: **S**uper-resolution **O**ptimized **U**sing **P**erceptual-tuned **G**enerative **A**dversarial **N**etwork (GAN), in order to produce thinner slices (e.g., higher resolution in the ‘Z’ plane) with anti-aliasing and deblurring. The proposed method outperforms other conventional resolution-enhancement methods and previous SR work on medical images based on both qualitative and quantitative comparisons. Moreover, we examine the model in terms of its generalization for arbitrarily user-selected SR ratios and imaging modalities. Our model shows promise as a novel 3D SR interpolation technique, providing potential applications for both clinical and research applications.

## 1. Introduction

Medical imaging modalities (e.g., MRI, CT, and ultrasound imaging) are widely used for clinical diagnosis and research [1]. They reveal internal anatomy and can provide quantitative measurements of the human body. Acquisition of a medical image depends on both the technical quality of the instrument and the conditions of the scan (e.g., patient properties, nature of the image being acquired), and in most cases, involves compromise, i.e., choosing a shorter acquisition time in MRI, reducing the radiation dose in CT, and using slower sweep speed in ultrasound. Thus, the acquired images may have low spatial resolution, typically in the ‘Z’ plane. For instance, an MRI using the 2D spin-echo technique can provide good in-plane resolution, but it typically has a high thickness for a clinically reasonable acquisition time, constraining the possibility of capturing important signals of lesions that are small compared to slice thickness. Moreover, the thick-slice images produce poor quality multiplanar or 3D reconstructions. There is active demand in practice to generate HR thin-slice images in order to reduce acquisition time, examination costs, and motion-induced artifacts.

One of the main approaches to accelerate MRI acquisition is through under-sampling in k-space, which inevitably results in aliasing artifacts by violating the Nyquist–Shannon sampling criteria [2], and then performing reconstruction of the under-sampled MRI, by solving an ill-posed inverse problem from an optimization point of view [3]. Currently, parallel imaging (PI) and compressed sensing (CS) [4] are widely used in clinical practice, and while the former method adopts multiple independent channels to simultaneously receive each view of the tissue area and utilizes software tools such as SENSE [5] or GRAPPA [6] to combine the multiple signals, the latter provides a framework for imaging reconstruction by adding a regularization term subject to prior sparsity constraints into the optimization problem. Beyond the conventional techniques, deep learning methods for MRI reconstruction have been extensively studied to learn the relationship between high-quality ground truths as target and the corresponding under-sampled images as input [7,8,9,10]. More advanced networks and algorithms are proposed following the idea of incorporating more “physics” from conventional methods (CS and PI), e.g., the development of variational networks [11,12,13]. In the case of CT, higher radiation dose is required to obtain thinner slices at a given noise level.

Single-image SR focuses on the 2D spatial domain and refers to the process of generating HR images from low-resolution (LR) images. Deep-learning-based SR models have been actively explored and they achieve state-of-the-art performance on natural image datasets [14,15,16,17,18]. One important breakthrough is the perceptual loss function [19,20,21], which may address the problem of blurring generated by the optimization of pixel-wise loss functions such as MSE. Together with GAN loss, perceptual loss has the potential to create images that are visually indistinguishable from the ground truth images.

Basic SR techniques have been applied to medical images [22]. Single-image methods using 2D convolutional neural networks (CNNs) are applied to CT in [23,24] and MRI in [25,26]. More advanced 2D models based on SRGAN with perceptual loss are investigated in [27,28]. SR using 3D CNNs are proposed for volumetric CT data [29,30,31]. For 3D SR MRI, Chaudhari et al. [32] implement CNNs to learn residual information between HR thin-slice images and LR thick-slice images using MSE loss. A self-supervised anti-aliasing algorithm is developed by [33,34] to resolve the LR out-of-plane slices by learning from the HR in-plane slices, thus requiring no external training dataset. High-quality results are reported by the SR MRI approaches in the literature [35,36,37,38,39]. However, there are certain limitations that preclude their application in real-world scenarios. Given the blurred textures produced by MSE loss, perceptual loss with GAN seems necessary in the single-image SR model, but it is unclear how to generalize a pre-trained 2D model based perceptual loss to 3D medical images. In modern medical-image deep-learning tasks, data augmentation is often applied during data preprocessing when the acquired data are insufficient. The adaptation of the SR MRI model to address a range of SR scenarios such as various sampling ratios, acquisition processes, or medical modalities is still limited. As a result, interpolation is still used instead of SR for routine data interpolation and augmentation.

In this study, we aimed to generate thinner SR of MR images from thicker (clinical) images. We propose a new algorithm termed SOUP-GAN: **S**uper-resolution **O**ptimized **U**sing a **P**erceptual-tuned **GAN** network for generating HR thin-slice images. Our main contributions are:

- We develop a scale-attention SR architecture that works for arbitrarily user-selected sampling factors with an overall satisfactory performance.
- We generalize the application of perceptual loss previously defined on 2D pre-trained VGG onto 3D medical images. Together with GAN, the new scheme with 3D perceptual loss significantly improves the perceived image quality over the MSE trained results.
- Two criteria of data preprocessing are proposed accounting for different acquisition protocols. Without examining the acquisition process carefully, the extracted datasets will not provide accurate mapping from LR slices into HR slices, and therefore may not work well for all the SR tasks.
- We evaluate the feasibility of designing one model that works for all the medical images. The proposed model is applied to datasets from other imaging modalities, e.g., T2-weighted MRI and CT, as well as many body parts.

Combining the above aspects, our model shows potential to be applied for the novel SR interpolations on medical images (Our SR interpolation tool based on SOUP-GAN is available at https://github.com/Mayo-Radiology-Informatics-Lab/SOUP-GAN, accessed on 11 March 2022).

## 2. Materials and Methods

### 2.1. Residual CNNs for 3D SR-MRI

Generally, image reconstruction is the transformation of signals acquired to images that are meaningful to humans and can be formulated as the multi-dimensional linear system:

(1) Ex=y+ε,

which tries to generate the desired images $x=\{x_{i},i\leq N\}$ based on the observed signals $y=\{y_{i},i\leq n\}$ with *N* and *n* as the numbers of desired and observed slices (images in the z-dimension), respectively. E is the encoder operator, representing various image generation or transformation tasks, e.g., an identity operator for image deblurring and denoising (with and without convolutions, respectively), image-space uniform under-sampling for SR, local masking operators for inpainting, and k-space under-samples for MRI reconstruction. ε is the coherent noise by the measurement.

SR tasks on 3D MRI specifically can be viewed as an under-sampling encoder trained to link HR slices x with LR slices y; while Equation (1) itself is ill-posed, it can be turned into an optimization problem:

(2) $$\hat{x}=\arg\min(\frac{1}{2}||Ex-{y||}_{2}^{2}),$$

and solved by supervised learning. Given a set of HR ground-truth examples x, the corresponding LR images y can be obtained through the encoder operator ${E=\left(x*\kappa \right)↓}_{s}$ that typically applies the anti-aliasing filtering κ with a subsequent down-sampling by the sampling factor *s* on the ground truth slices. The training dataset is composed of the ground-truths x and their associated under-sampled y, on which a generator network $G_{\theta }$ is proposed and trained to approximate the function F, the inverse form of the encoder E, such that x=F(y).

The designed generator $G_{\theta }$ consists of a pre-upsampling layer by cubic interpolation together with a residual network of 3D convolutional layers, or 3D U-net, on which the data patches can be efficiently trained. For the residual learning, cubic interpolation can better approximate the ground truth compared to lower-order interpolations, and the number of convolutional layers is selected as 14, taking into account both performance and computational effort. For the task involving a large training dataset, the so-called RRDB (Residual in Residual Dense Block) introduced in ESRGAN [18] is adopted to better resolve the residual spatial details, and to alleviate the burden of occupied memory through such a post-upsampling scheme.

**Figure 1:**
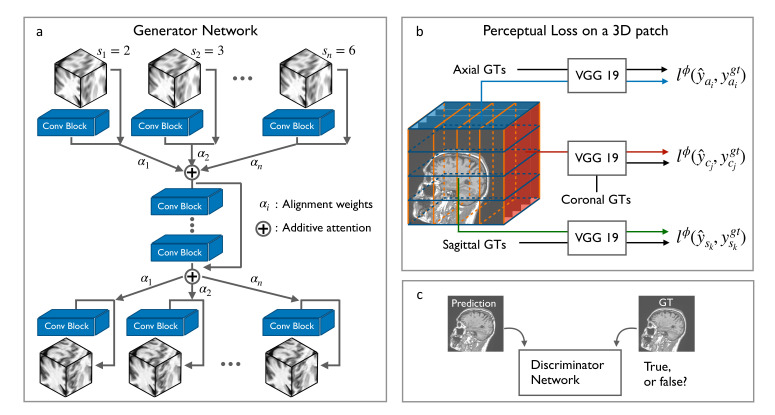
(**a**) Architecture of our scale-attention SR network with GAN. (**b**) Three-dimensional perceptual loss is calculated by inputting all the axial, sagittal, and coronal surfaces of the 3D data into the pre-trained VGG and (**c**) comparing the differences between predictions and ground-truths in the high-dimensional feature space. GT: ground truth.

As an outline of the proposed framework SOUP-GAN, Algorithm 1 is provided with pseudocode to summarize the training process including multiple processing steps, which are depicted by Figure 1 and explained in the later subsections.
**Algorithm 1** Training the proposed framework SOUP-GAN.**Data:** HR 3D MRI volume after data standardization.**Step** (1) **Data preprocessing:** Create LR volume by data-preprocessing following either the *thick-to-thin* or *sparse-to-thin* criteria.**Step** (2) **Prepare training dataset:** Partition the LR data as input and HR data as ground truth into pairs of 32×32×32 patches.**Step** (3) **Scale-attention SR network:** Input LR patches to the attention-based multi-scale SR network with an appropriate module entrance and exit by the calculated alignment weights α according to the associated sampling factor *s*.**Step** (4) **3D perceptual loss with GAN:** Based on the pre-trained MSE results, further tune the model by employing the 3D perceptual loss with GAN.

### 2.2. Data Preprocessing

IRB reviewed this protocol and waived the requirement for patient consent. Data acquired for this study are 3D MRI datasets. One hundred T1-weighted brain MRI scans obtained at our hospital in 2018 and 2019 for multiple sclerosis (MS) were collected. Those are HR thin slices (256×256×160) with 1 mm isotropic pixel spacing in both the in-plane and the out-of-plane directions. The LR volumes were created by the data-preprocessing methods explained later. These 100 scans were distributed into training, validation and test datasets at a ratio of 8:1:1 at the patient level (i.e., all patches from a given patient were only in one of the groups). Both the input and ground-truth datasets were then cropped and partitioned into pairs of patches; while larger volumetric patches potentially capture better semantic features [40], we selected a patch size of 32×32×32 to reduce computational demands. All patch pairs were shuffled within each group to avoid possible data leakage among training, validation, and test. Other 100 T2-FLAIR brain MRI scans were collected for evaluating the generalization of our SR model to other contrast types of MRI data.

The specific way to prepare LR images from HR images depends on the acquisition protocols and the up-sampling factor *s* selected for the SR task. We define two different criteria accounting for the situations broadly categorized in the acquisition process, because without taking care of these variations, the extracted datasets will not provide accurate mapping from LR slices back to HR slices, and therefore will fail to work well for various resolution-enhanced tasks in practice. In detail, the preprocessing from HR ground-truth data to the associated LR representation is categorized into two types: (1) *thick-to-thin* and (2) *sparse-to-thin*.

The first type (*thick-to-thin*) assumes the same FOV of the acquired 3D data (i.e., same Z coverage). The selection of the slice thickness determines how much tissue is to be covered within each slice. Thicker slices can achieve more efficient acquisition without slice gaps or spatial aliasing but they cause image blurring due to partial volume artifact. Correspondingly, the thick-slice representation of the HR ground-truth data is obtained by the finite impulse response (FIR), low-pass filtering with the filter coefficients as b=1/s to mimic the averaging and anti-aliasing characteristic with the high thickness, followed by a subsequent down-sampling with the factor *s*.

The second type (*sparse-to-thin*) simulates scans that are expedited by discretizing FOV in Z (i.e., same 2D X-Y resolution). This is useful for acquisition protocols focused on the in-plane resolution without considering the perpendicular (Z) planes. Thin slices with large separation are acquired efficiently. As a result, they are insufficient to cover the whole FOV in Z direction, therefore inevitably introducing spatial aliasing in the perpendicular planes [41]. One clinical example is for diagnosing interstitial lung disease in which HR CT data (∼1 mm slice thickness) are acquired at 10 mm intervals. Corresponding to this type of clinical acquisition as well as imaging interpolation in deep learning tasks, the LR representation of the HR ground-truth data is obtained directly by the down-sampling with the factor *s*.

For other cases between the first and second types of data preprocessing discussed here, a Gaussian filter with a tuned value of the standard deviation can be adopted. The LR representation obtained by either the *thick-to-thin* or the *sparse-to-thin* criteria is then used to prepare the input dataset for the SR network.

### 2.3. Scale-Attention Model for SR Interpolation

Supervised medical deep-learning tasks (e.g., classification, detection, and segmentation) are based on the acquired images and associated labels. The accuracy of the model inference is not only decided by the design of the networks, but more importantly it relies on the data size and quality. Data augmentation is often performed when the training data are insufficient, imbalanced, or incompatible in shape. Conventional interpolations (such as linear, quadratic, and cubic) are widely used and can provide approximate intermediate slices. However, when the sampling factor *s* increases, the interpolated new images become more blurred. From our observation on SR tasks, we design an attention-based multi-scale model to provide image interpolation with superior resolution, upon arbitrarily user-selected sampling factor *s*.

**Figure 2:**
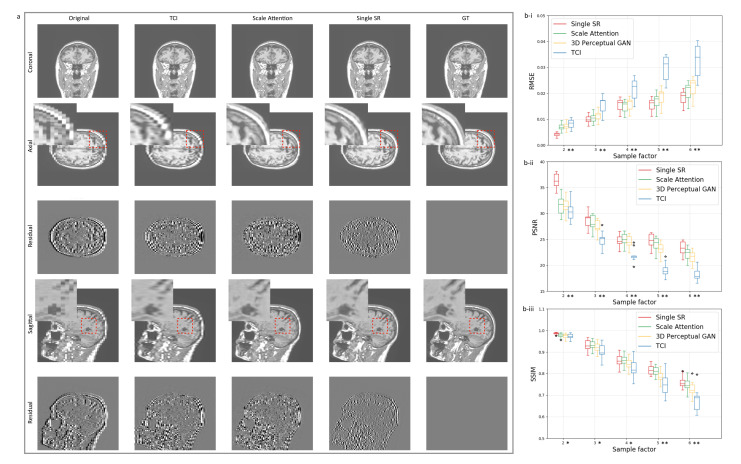
(**a**) Comparison between single-scale model and scale-attention model on T1-weighted brain MRI. The figure shows original slices, cubic interpolation and ground-truth slices as a reference. Residual images at higher contrast are added to better visualize the difference of the generated images to the ground-truths. (**b**) Quantitative comparisons of measures: (**b-i**) RMSE, (**b-ii**) PSNR, and (**b-iii**) SSIM in terms of the sampling factor s between the single-scale model, the scale-attention model, 3D perceptual GAN (SOUP), and cubic interpolation. Statistical significance is indicated on the x-axes with * for p<0.05 and ** for p<0.001. TCI: tricubic interpolation; GT: ground truth.

In our scale-attention architecture, the single-scale model described earlier provides the starting point for learning the residual between LR and HR slices. Pre- and post- attention modules are added, respectively, at the top and the bottom layers, to deal with the differential information between scales. As a result, we construct one model based on selected sampling factors $s_{i}\in \left\{2,3,4,5,6\right\}$ that can provide predictions for general cases with sampling factors ranging from 1.5 to 6.5 to cover most usual needs in practice (see Figure 1 and Figure 2b), while most of the model parameters are shared by the backbone across the scales.

For a user-selected sampling factor *s*, we incorporate it to the attention model by calculating the alignment weights:

(3) $$\alpha =\left\{\begin{array}{cc} {}[1,0,\ldots ,0], & s<2\\ {}[0,\ldots ,\left(1-t\right),t,\ldots ,0], & m\leq s<m+1\\ {}[0,\ldots ,0,1], & s\geq 6 \end{array}\right.$$

in which the terms (1−t) and *t* correspond to the elements $\alpha_{m-1}$ and $\alpha_{m}$, respectively, for any fractional *s* between the integer *m* and m+1 with the fraction t=s−m. The attention-layer output is then an α weighted sum of all the scale branches: $\sum \alpha_{i}y_{i}$. The proposed scale-attention SR model provides an efficient tool to perform the image interpolation with higher resolutions than conventional interpolation methods.

### 2.4. 3D Perceptual Loss

In previous single-image SR tasks studied on natural images, it was observed that training based on a pixel-wise loss, e.g., L1, MSE, or PSNR can achieve relatively high metric scores, but often results in overly-smooth SR images with blurring, since the perceptual quality of textures, edges, and high-frequency details are ignored by the pixel-wise losses. Based on the idea of being closer to semantic similarity, Gatys et al. [19] propose perceptual loss, which is defined by evaluating the Euclidean distances between the feature maps extracted from a pre-trained VGG network instead of low-level pixel-wise error. For instance, perceptual loss based on the high-level representations at the *l*-th layer of the VGG19 network [42] is formulated as:

(4) $$L_{per}=\frac{1}{hwc}\sum_{i,j,k}{\left(\phi_{i,j,k}^{l}\left(x\right)-\phi_{i,j,k}^{l}\left(F\left(y\right)\right)\right)}^{2},$$

where *h*, *w*, and *c* are the height, width, and channel number of the representation on the layer *l*, respectively.

It is worth noting that SR MRI tasks deal with 3D medical images, while the VGG network takes 2D images as input. To generalize the single image SR with perceptual loss into 3D, we propose to adopt the original slices together with adjacent planes of the ground truth volume *x* and the predicted volume F(y). That is, all the axial, sagittal, and coronal images of the 3D data are imported into the pre-trained VGG and contribute to the perceptual loss calculation (see Figure 1). In this way, the 3D MRI volume is perceptually resolved in terms of projected slices along the three principal directions. We also note that we used the deep intermediate layer VGG19-54 before activation rather than after activation.

In addition to the perceptual loss, we define a discriminator network $D_{\theta }$, together with the generator model $G_{\theta }$, based on the generative adversarial networks structure. The generator $G_{\theta }$ and discriminator $D_{\theta }$ are optimized in an alternating manner to solve the adversarial min–max problem:

(5) $$\min_{G}\max_{D}E_{x∼p_{\mathrm{gt}}}\left[\log D\left(x\right)\right]+E_{y∼p_{\mathrm{train}}}\left[\log\left(1-D\left(G\left(y\right)\right)\right)\right],$$

which aims to train a generative model $G_{\theta }$ trying to fool the discriminator $D_{\theta }$, while the discriminator is trained simultaneously to distinguish the generated SR images from ground truth images. With this approach of adversarial training between $G_{\theta }$ and $D_{\theta }$, the generated images are encouraged to reside in the featured manifold of real images, potentially with perceptually superior quality that is visually indistinguishable from the ground truth images. Other options in the literature for the GAN stage include analyzing features and graph properties extracted from the image, e.g., Markov random fields (MRFs) used for texture synthesis and inpainting [43,44].

## 3. Results

### 3.1. Training Details

The training process is divided into two stages. The generator model $G_{\theta }$ is first trained by the MSE loss. The Adam optimization is used. The learning rate is initialized as $3\times 10^{-4}$, and decreased by a factor of 3 upon three cycles, if the validation loss is no longer updated for the current cycle. Then, the model is further tuned by employing the perceptual loss and GAN. The total loss for $G_{\theta }$ upon the refined tuning is:

(6) $$L_{G}=L_{per}+\lambda L_{GAN}+\mu L_{MSE},$$

where the weights λ and μ are the coefficients to balance different loss terms and set as 0.01 and 0.001 [18]. The pre-trained pixel-wise model is used to initialize the second stage of training. Transfer learning helps the perceptual-tuned GAN model converge more efficiently and obtain more visually pleasing results. The generator $G_{\theta }$ and the discriminator $D_{\theta }$ are alternately updated until the model converges to produce visually satisfactory images and no further improvement is visually apparent. We recognize that this is an arbitrary criterion, but human perception is the desired application and using perceptual metrics would also be weak since they are used to train the model.

### 3.2. Single-Scale and Scale-Attention Model Comparison

The SR technique shows its potential as a better “interpolation”, given the active demand for data augmentation in medical-imaging deep-learning tasks. The feasibility of this idea requires an adaptive SR model which is designed to incorporate random sampling factors. Figure 2a compares the single-scale SR model with our scale-attention SR model at a sampling factor equal to 5. We can imagine that, to some extent, the scale-attention model must sacrifice partial resolutions, as a trade-off for allowing arbitrary scale entries. However, the images obtained by the scale-attention model are still of satisfactory quality compared to the tricubic interpolation (TCI) and single-scale model.

**Figure 3:**
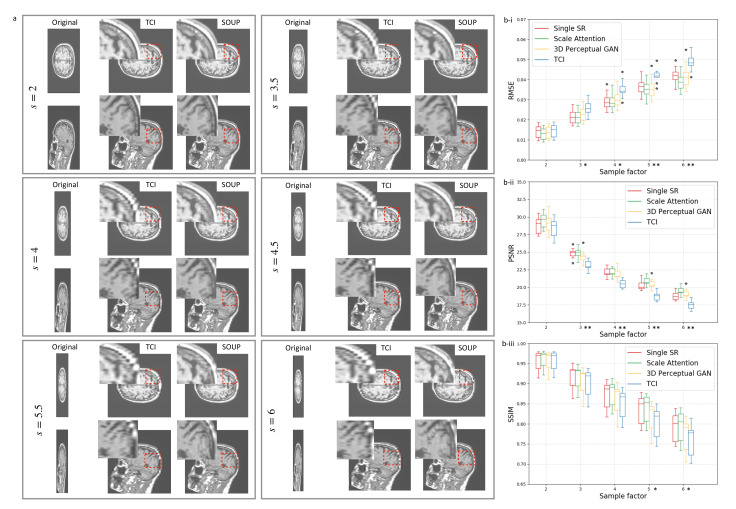
(**a**) Comparison between scale-attention model and cubic interpolation at different scales. (**b**) T1-weighted brain MRI test dataset with quantitative comparisons of measures: (**b-i**) RMSE, (**b-ii**) PSNR, and (**b-iii**) SSIM in terms of the sampling factor s. As a reference, DeepResolve proposed by [32] as a single-scale residual-based 3D CNNs with MSE is similar to the single SR reported here (in red). Statistical significance is indicated on the x-axes with * for p<0.05 and ** for p<0.001. TCI: tricubic interpolation; GT: ground truth.

Beyond the single-scale limit, our scale-attention model is demonstrated to work for user-selected scale values, which is the key to turning the SR model into a useful interpolation tool. Figure 3b reports the quantitative data performed on T1-weighted brain MRI test dataset with the metrics at different scales, including the root of mean squared error (RMSE), peak signal to noise ratio (PSNR), and the structural similarity (SSIM). Figure 3a shows the generated images at different scales, obtained by SOUP (the scale-attention model) and compared to the conventional interpolation results. As a summary of the comparison, while the scale-attention SR model and the conventional interpolation method converge to a similar quality at the scale s≤2, the SR model starts to outperform the interpolation and achieves better and better image qualities when the scale *s* increases.

### 3.3. Application to Other Contrast Types of MRI Images

**Figure 4:**
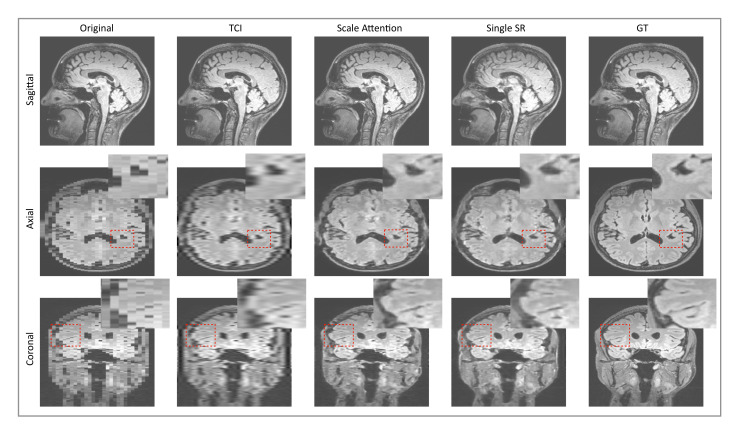
Comparison between the scale-attention model and cubic interpolation on T2-FLAIR brain MRI at different scales. TCI: tricubic interpolation; GT: ground truth.

**Figure 5:**
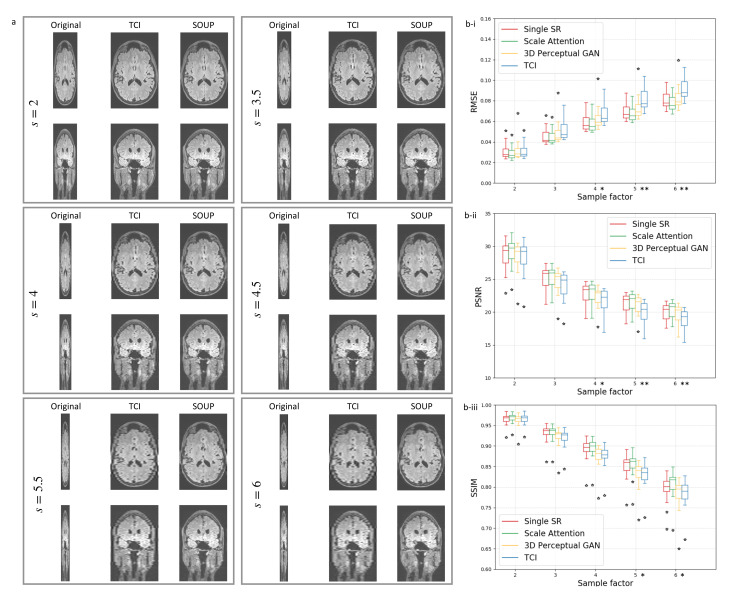
(**a**) Comparison between scale-attention model and cubic interpolation on T2-FLAIR brain MRI at different scales. (**b**) Evaluation using T2-weighted brain MRI images with quantitative comparisons of measures: (**b-i**) RMSE, (**b-ii**) PSNR, and (**b-iii**) SSIM in terms of the sampling factor s. DeepResolve proposed in [32] is similar to the single SR (in red). Statistical significance is indicated on the x-axes with * for p<0.05 and ** for p<0.001. TCI: tricubic interpolation; GT: ground truth.

In the literature, single-image SR models are trained and tested on datasets of natural images, collected from different categories with various distributions. An open question for medical images is whether it is possible to develop a generalized SR model that works for various medical image data types (e.g., modalities or contrast types) with an arbitrary sampling factor *s* selected by the user. To address this question, we apply our scale-attention SR model to the test datasets of other medical imaging types, e.g., T2-FLAIR MRI and CT, and other body parts. Figure 4 shows the comparison between the single-scale model and the multi-scale model on FLAIR brain MRI, Figure 5b quantitatively evaluates the performances of the models on the FLAIR MRI dataset with different metrics, and Figure 5a compares SOUP with conventionally interpolated images at a different sampling factor *s*.

### 3.4. Generalization to Other Medical Imaging Modalities, e.g., CT

**Figure 6:**
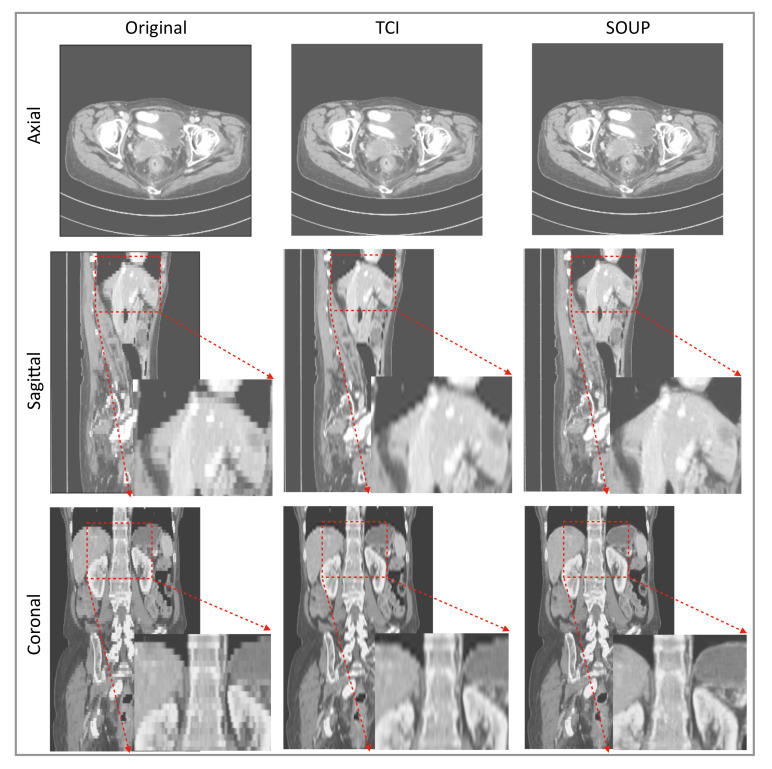
Example of SR interpolation on CT with a sampling factor equal to 4.8. Edges and textual details are better resolved by SR. Blurring is reduced. TCI: tricubic interpolation.

**Figure 7:**
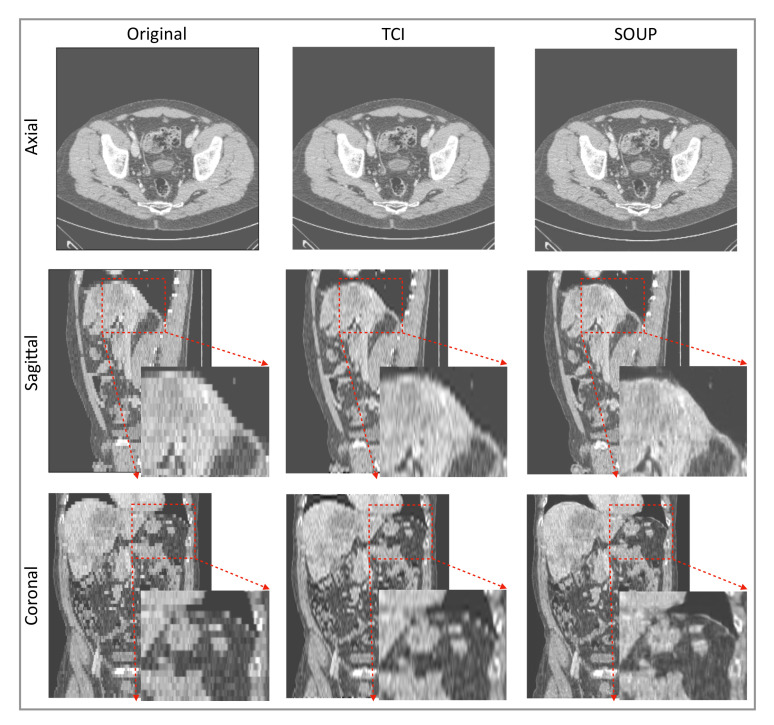
Another example of SR interpolation on CT data with a sampling factor equal to 5.72. TCI: tricubic interpolation.

We also apply our scale-attention SR model to CT data. Figure 6 and Figure 7 show the SR-interpolated (SOUP) and conventionally interpolated images on abdominal and pelvic CTs. It is clear from both examples that the SR-interpolated results achieve significantly higher quality than the conventionally interpolated ones in regard to the edges, textual details, and blurring. Those comparisons demonstrate that our scale-attention SR model (SOUP) is a widely generalizable tool for different sampling factors and various applied imaging modalities, which paves the way for more advanced medical image interpolation through deep-learning SR.

## 4. Discussion

Data interpolation is important in medical imaging and deep-learning tasks. Data preprocessing through mathematical interpolations is often used when the training data are insufficient, imbalanced, or incompatible in shape. Though SR approaches can generate images with better quality, interpolation is still used instead of SR for routine interpolation. A significant limitation of prior SR methods, including the multi-scale SR first proposed by [17], is that they are designed to work only for a fixed and pre-defined sampling ratio; they therefore fail to adapt to arbitrarily user-selected ratios. Figure 3 and Figure 5 show the flexibility of our approach to deal with variable ratios. For realistic applications of SR interpolation, we proposed two inference models based on two different types of data preprocessing: the thick-to-thin model is used for interpolating 3D images without slice gaps, and the sparse-to-thin model is for interpolating sparsely stacked 2D images.

**Figure 8:**
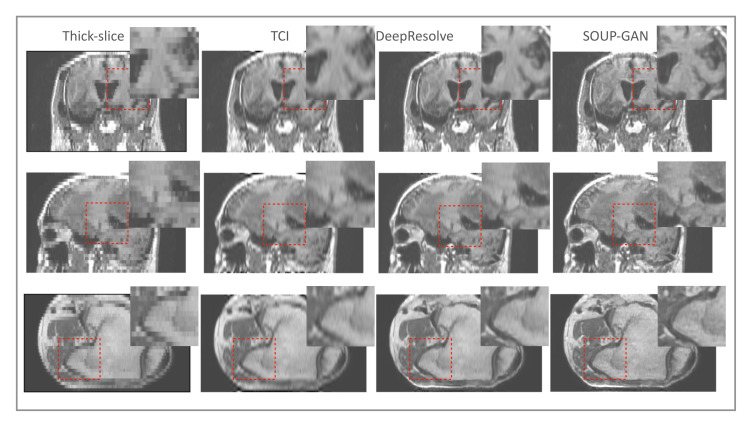
Ablation study: Test examples on brain and knee MRI showing perpendicular reconstructions from original thick slices, using TCI, MSE, and GAN using 3D perceptual loss (SOUP). We note that [32] proposed a single-scale residual-based 3D convolutional neural network with MSE, similar to the third column here. Our SOUP approach better resolves the perceptual details and is more generally applicable to various sampling factors and different imaging modalities. TCI: tricubic interpolation.

As discussed in Section 2, pixel-wise loss usually achieves a relatively high metric score but fails in resolving the textures, edges, and high-frequency details, resulting in blurring of the images that are produced. The scheme that we propose generalizes the application of perceptual loss previously defined on 2D pre-trained VGG to study single-image SR onto 3D medical images. Through this simple but effective idea of importing all the projected slices along the three principal directions into the pre-trained VGG, we observed that the 3D MRI volume can be perceptually resolved (see the ablation study Figure 8). SOUP-GAN was compared to another MRI SR approach called “DeepResolve”, both of which focus on thick-slice to thin-slice reconstruction. The two test datasets included in Figure 8 are the T1-weighted brain MRI (reformatted coronal images in the first row, and reformatted sagittal images in the second), and knee MRI in the third row. The images appear better compared to the original thick slices, the cubic-spline interpolated images, and the MSE results. They are further improved by 3D perceptual loss with GAN, achieving the highest perceptual quality. Besides the three principal directions (e.g., axial, sagittal, and coronal), we noted that oblique planes are also able to be resolved through multi-planar reconstruction with our 3D perceptual-loss strategy (unpublished data). We used a ’vanilla’ GAN instead of more advanced GANs, e.g., Wasserstein [45] or Relativistic GAN [46], since the vanilla GAN performs well for our training.

It was encouraging to see that our model, which was trained on MRI T1 brain data, generalized to other contrast types of MRI and CT data, and other body parts. Rather than training a different specialized model for each medical modality and body part, it is more convenient to design one model that works for all medical images to achieve SR interpolation. CT data expressed in Hounsfield units obtained from a linear transformation of the measured attenuation coefficients have different distributions from MRI data. We note here that data standardization by scaling them into a uniform range, e.g., (0, 10,000), was necessary to make the SR model compatible for different imaging modalities. Although less accurate than training a model directly on CT data, Figure 6 and Figure 7 show good performance using our T1-trained model for CT data inference. Since they are realistic applications of SR interpolation performed on poor quality data (generating thin slices from thick slices), no ground-truth data is available to calculate metrics of generated images.

## 5. Conclusions

We combined a deep-learning SR method with a 3D perceptual-tuned GAN network, and examined its application and generalization in terms of arbitrary sampling scales, various medical imaging modalities, and different body parts, as a novel 3D image interpolation technique. The SR thin slices obtained by the model outperform other conventional resolution-enhancement methods and previous SR work on medical images based on both qualitative and quantitative comparisons. Based on the promising simulation data, the proposed work shows potential in both clinical tasks, such as reducing the acquisition time and further resolving the MRI scans in retrospective studies, and research tasks, e.g., serving as a novel SR interpolation method for data augmentations with further applications in lesion measurements, radiomics, and automatic segmentations.

## 6. Patents

A patent “Systems and methods for deep learning-based super-resolution medical imaging.” with application No. 63/230,607 (Mayo Clinic: 2020-500) was filed on 08/06/2021.

## Data Availability

Super-resolution interpolation tool for medical images based on the proposed SOUP-GAN model is available at https://github.com/Mayo-Radiology-Informatics-Lab/SOUP-GAN, accessed on 11 March 2022. The provided package supports thick-slices to thin-slices SR interpolation with arbitrarily user-selected sampling ratios (e.g., s∈[1.5,6.5]).
